# High intake of vegetables is linked to lower white blood cell profile and the effect is mediated by the gut microbiome

**DOI:** 10.1186/s12916-021-01913-w

**Published:** 2021-02-11

**Authors:** Cristina Menni, Panayiotis Louca, Sarah E. Berry, Amrita Vijay, Stuart Astbury, Emily R. Leeming, Rachel Gibson, Francesco Asnicar, Gianmarco Piccinno, Jonathan Wolf, Richard Davies, Massimo Mangino, Nicola Segata, Tim D. Spector, Ana M. Valdes

**Affiliations:** 1 Department of Twin Research and Genetic Epidemiology, King’s College London, St Thomas’ Hospital Campus, Westminster Bridge Road, London, SE1 7EH UK; 2 Department of Nutritional Sciences, King’s College London, Franklin-Wilkins Building, Stamford St, London, SE1 9NH UK; 3 School of Medicine, University of Nottingham, Academic Rheumatology Clinical Sciences Building, Nottingham City Hospital, Hucknall Road, Nottingham, NG5 1PB UK; 4 National Institute for Health Research (NIHR) Nottingham Biomedical Research Centre, Nottingham University Hospitals NHS Trust and the University of Nottingham, Nottingham, UK; 5 Department CIBIO, University of Trento, Via Sommarive 9, 38123 Povo, Trento, Italy; 6 Zoe Global Ltd, 164 Westminster Bridge Rd, Bishop’s, London, SE1 7RW UK; 7 NIHR Biomedical Research Centre at Guy’s and St Thomas’ Foundation Trust, London, SE1 9RT UK

**Keywords:** White blood cell, Gut microbiome, Diet, Vegetable intake, Chronic inflammation

## Abstract

**Background:**

Chronic inflammation, which can be modulated by diet, is linked to high white blood cell counts and correlates with higher cardiometabolic risk and risk of more severe infections, as in the case of COVID-19.

**Methods:**

Here, we assessed the association between white blood cell profile (lymphocytes, basophils, eosinophils, neutrophils, monocytes and total white blood cells) as markers of chronic inflammation, habitual diet and gut microbiome composition (determined by sequencing of the 16S RNA) in 986 healthy individuals from the PREDICT-1 nutritional intervention study. We then investigated whether the gut microbiome mediates part of the benefits of vegetable intake on lymphocyte counts.

**Results:**

Higher levels of white blood cells, lymphocytes and basophils were all significantly correlated with lower habitual intake of vegetables, with vegetable intake explaining between 3.59 and 6.58% of variation in white blood cells after adjusting for covariates and multiple testing using false discovery rate (*q* < 0.1). No such association was seen with fruit intake. A mediation analysis found that 20.00% of the effect of vegetable intake on lymphocyte counts was mediated by one bacterial genus, *Collinsella*, known to increase with the intake of processed foods and previously associated with fatty liver disease. We further correlated white blood cells to other inflammatory markers including IL6 and GlycA, fasting and post-prandial glucose levels and found a significant relationship between inflammation and diet.

**Conclusion:**

A habitual diet high in vegetables, but not fruits, is linked to a lower inflammatory profile for white blood cells, and a fifth of the effect is mediated by the genus *Collinsella*.

**Trial registration:**

The ClinicalTrials.gov registration identifier is NCT03479866.

**Supplementary Information:**

The online version contains supplementary material available at 10.1186/s12916-021-01913-w.

## Background

Inflammation is a normal component of host defence; however, elevated unresolved chronic inflammation is a core perturbation in a range of chronic diseases [1]. Chronic inflammation and activation of immune cells are key mechanisms in the development of atherosclerosis with hypercholesterolemia-induced neutrophil recruitment promoting early atherosclerotic changes [2]. Moreover, inflammation is an important contributor to type 2 diabetes, via the processes of insulin resistance and islet β cell failure [3], and increased white blood cell (WBC) counts are predictive of incidence for type 2 diabetes [3, 4]. Neutrophils, which are the predominant circulating WBC in humans, kill and degrade microbes via the process of phagocytosis [5] and are major effectors of acute inflammation. They also contribute to chronic inflammatory conditions [6] including atherosclerosis [2] and adipose tissue inflammation [7]. Furthermore, chronic inflammation promotes lymphocyte infiltration into inflamed non-lymphoid tissues that do not recruit significant numbers of lymphocytes under normal conditions [8].

In the past decade, the composition of the gut microbiome has been identified as a key modifier of chronic inflammation and cardiometabolic risk [9]. Interactions between toll-like receptors (TLRs) and NOD-like receptors (NLRs) of the intestinal epithelium and bacterial pathogen-associated molecular patterns (PAMPs), the most common being lipopolysaccharide present on the membrane of Gram-negative bacteria, can result in the release of proinflammatory cytokines. This results in a balance between regulation of expression of epithelial receptors in the gut and stimulation by the gut microbiota. Independently of this direct interaction between the gastrointestinal epithelium and constituents of the microbiota, by-products of bacterial fermentation of components of the diet can also modulate the immune system. For example, short chain fatty acids produced via the fermentation of fibre will inhibit inflammation via suppression of monocyte and macrophage recruitment and cytokine production [10]. Conversely, trimethylamine-N-oxide, formed through the microbial breakdown of choline to trimethylamine after metabolism in the liver, will induce inflammation and atherosclerosis via inhibition of cholesterol transport and promotion of macrophage cholesterol accumulation by modulating scavenger receptor expression [11, 12]. As the main site of nutrient absorption, there is a clear interdependence between the gut microbiome and diet, with both of these factors interacting with the immune system [13].

A substantial amount of evidence suggests that many foods, nutrients and non-nutrient components modulate inflammation and immune function, both acutely and chronically [14–16]. In particular, a reduction in inflammatory markers (hs-CRP and TGF-ß) has been observed in individuals adhering to a healthy plant-based diet [17, 18]. More specifically, a role has been proposed for dietary nitrate, the main source of which is leafy green vegetables such as spinach and lettuce [19] . In a 12-week placebo-controlled animal model, nitrate-fed mice had reduced systemic leukocyte rolling and adherence, circulating neutrophil numbers, neutrophil CD11b expression and myeloperoxidase activity (an enzymatic marker of neutrophil tissue infiltration, compared with wild-type littermates) [20]. Additionally, dietary nitrate administration was reported to reduce tissue level expression of myeloperoxidase [21] and oxidative stress [22], a pathophysiological process closely related to chronic low-grade inflammation [23].

The COVID-19 pandemic [24] has revealed a significantly higher risk of hospitalisation and death among individuals with cardiometabolic comorbidities, including type 2 diabetes, hypertension and cardiovascular diseases [25], with associated endothelial dysfunction [26]. This has brought to the forefront the key question of how can inflammatory markers be modulated by diet and what are the links between diet, gut microbiome and inflammation.

The aim of this study is to investigate the links between white blood cell counts (overall and by subtypes), as markers of chronic inflammation, habitual diet and gut microbiome composition in the PREDICT-1 Study [27].

## Methods

### Study design and participants

We included 986 individuals from the UK-based PREDICT-1 study with 16S gut microbiome, white blood cell profile markers and who completed a food frequency questionnaire (FFQ). The PREDICT-1 study [27] was a single-arm nutritional intervention conducted between June 2018 and May 2019. Study participants were healthy individuals (thus eliminating potential confounders brought about by the presence of infections or other comorbidities) aged between 18 and 65 years recruited from the TwinsUK registry [28] and the general population using online advertising. Participants attended a full day clinical visit consisting of test meal challenges followed by a 13-day home-based phase, as previously described [27].

#### Blood cell count measurements

Blood samples were collected using EDTA tubes for measurement of the complete blood cell counts. They were analysed on a blood cell counter (Beckman Coulter, CA). The following parameters were considered as exposures: WBC; total and differential WBC count (109 cells/L), including lymphocytes, monocytes, neutrophils, eosinophils and basophils.

#### Inflammatory markers

Levels of IL-6 were measured by Affinity Biomarkers, London, using the standardised Human Proinflammatory panel 1 assay kit (cat number K151A0H-1), distributed by Meso Scale Discovery.

#### Dietary information

Habitual dietary information was estimated via food frequency questionnaires (FFQs), and nutrient intakes were determined using FETA software to calculate macro- and micronutrient [29]. FFQs were excluded if more than 10 of 187 food items were left unanswered or if the estimated total energy intake derived from FFQ as a ratio of the subject’s estimated basal metabolic rate (determined by the Harris–Benedict equation) [29] was more than 2 standard deviations outside the mean of this ratio (< 0.52 or > 2.58), as previously described [27]. From FFQs, intake for fruit and vegetables was quantified by the number of portions and grams. In addition to total vegetable intake, 5 categories of vegetables were generated from characteristics, including allium, cruciferous, green leafy, yellow and other (as explained in Table S1).

*Nitrate intake* was estimated from 19 vegetables that overlapped with the European Food Safety Authority panel on nitrates in vegetables [19], where European member states were requested to report nitrate concentrations in individual vegetable samples. To ensure comparable levels of performance between laboratories, strict requirements to commission regulation (No. 1882/2006) were in place. Over 21 states provided 40,861 data points [19]. Mean (mg/kg) and median (mg/kg) nitrate concentrations for each type of vegetable were then estimated [19], facilitating nitrate content (mg) per portion for 19 vegetables from our FFQ to be calculated. For each vegetable, nitrate per portion was multiplied by reported quantity consumed. Then, nitrate intake from all 19 vegetables was summated to generate total nitrate intake.

#### The plant-based diet index

Two versions of the plant-based diet index were considered [30]: the healthy plant-based index (h-PDI) and the unhealthy plant-based index (u-PDI). Eighteen food groups (amalgamated from the FFQ food groups) were assigned either positive or reverse scores after segregation into quintiles [31]. For the h-PDI, positive scores were applied to the ‘healthy’ plant foods, with a score of 5 given to those in the highest quintile and 1 in the lowest, and a reverse score to the ‘less-healthy’ plant foods and the animal-based foods. Scores were then summated with possible scores ranging from 18 to 90 (18 food groups scored at a minimum 1 and maximum 5). The opposite arrangement was applied to the u-PDI, a positive score to ‘less-healthy’ and a negative score to ‘healthy’ plant foods.

### Stool-sample collection

Participants collected a stool sample at home prior to their clinical visit using the EasySampler collection kit (ALPCO). Upon receipt at the laboratory, samples were homogenised, aliquoted and stored at − 80 °C in Qiagen PowerBeads 1.5-ml tubes (Qiagen) as previously described [27].

### Microbiome 16S RNA gene sequencing

Gut microbiome composition was determined by 16S rRNA gene sequencing carried out as previously described [27]. Briefly, the V4 region of the 16S rRNA gene was amplified and sequenced Genomescan. Quality control of the reads was carried out using the ‘filterAndTrim’ function from the DADA2 package, truncating eight nucleotides from each read to remove barcodes, discarding all reads with quality less than 20, discarding all reads with at least one N and removing the phiX Illumina spike-in. Only paired-end reads with at least 120 bp and with an expected DADA2 error less than 4 were retained for downstream analyses. Merged reads were further processed, and only reads within 280 and 290 bp were retained, representing the majority of the distribution of the lengths. Reads were further processed to remove chimeras. Finally, taxonomy was assigned using the SILVA database (version 132) using the ‘assignTaxonomy’ function, requiring a minimum bootstrap value of 80, to obtain a table of relative abundances of operational taxonomic units. The relative abundance values were normalised using the arcsine square root transformation as described elsewhere [27]. The arcsine square root transformation is a monotonic transformation useful for improving normality as the variance of the distribution results more stable and has been previously used in finding associations using a general linear model [32] and for detecting a microbial signature in colorectal cancer patients [33].

### Lipoprotein profiling by nuclear magnetic resonance

Circulating levels of triglyceride (TG) and glycoprotein A (GlycA) were measured by Nightingale Ltd. (previously known as Brainshake Ltd., Finland, https://www.brainshake.fi/) from fasting serum samples using 500 Mhz proton nuclear magnetic resonance spectroscopy as previously described [34].

### Statistical analysis

Statistical analysis was carried out using Stata version 12.

We ran linear regression to evaluate the associations between white blood cell counts and (i) fruit and vegetable; (ii) microbiome abundances; (iii) markers of inflammation; and (iv) cardiometabolic phenotypes intake, adjusting for age, gender, BMI and multiple testing using false discovery rate (*q* < 0.1). All traits were standardised to have mean 0 and 1 SD to allow effect comparison across traits.

We used all OTUs that were present in at least 70% of all participants (*n* = 690). This resulted in a total of 85 OTUs with a mean relative abundance of 1.1%. Using this cut-off to achieve a *q* < 0.1 allows to test for 510 independent tests (85 OTUs × 6 traits) with a nominal alpha of 0.00019. This sample size and the cut-off used have 80% statistical power to observe correlations between OTU relative abundances and the six traits investigated with *r* = 0.17 or higher.

We further employed mediation analysis as implemented in the Stata package ‘medeff’ to test the mediation effects of gut microbiome diversity (indirect effect) on (i) the total effect of vegetable intake on white blood cells (lymphocytes); (ii) the total effect of dietary nitrate on white blood cells (lymphocytes), adjusting for age, gender and BMI. We further adjusted for family relatedness. A mediation model identifies the mechanism underlying an observed relationship between an independent variable (X- here vegetable intake or nitrate intake) and a dependent variable (Y- here lymphocytes) via the inclusion of the mediator variable (M- here gut microbiome diversity). Rather than a direct causal relationship between vegetable intake/nitrate and lymphocytes, a mediation model proposes that the independent variable (vegetable intake/nitrate) influences the mediator variable gut microbiome diversity, which in turn influences the dependent variable lymphocytes. As such, the mediation model provides greater understanding between the relationship between an independent variable and a dependent variable when these variables do not have an obvious direct connection. We constructed a mediation model to quantify both the direct effect of vegetable intake/dietary nitrate on white blood cells and the indirect (mediated) effects mentioned above. A full mediation is indicated in the case where the direct effect *c’* is not significant, whereas the indirect effect *a × b* is significant. This means only the indirect effect via the mediator exists. All other situations under the condition that both the direct effect *c’* and the indirect effect *a × b* are significant represent partial mediation [35].

The variance accounted for (VAF) score, which represents the ratio of indirect-to-total effect and determines the proportion of the variance explained by the mediation process, was further used to determine the magnitude of the mediation effect [36]. A VAF exceeding 80% supports an additional argument for a full mediation [35].

## Results

The descriptive characteristics of the study population are presented in Table 1. Of the 1002 PREDICT-1 participants [27], here we included 986 individuals (of which 246 twin pairs) with 16S gut microbiome, white blood cell profile markers and who completed an FFQ. The average energy intake of the included subjects was 2345.97 Kcal and comprised of 262.36 g carbohydrates, 109.19 g fat and 80.75 g protein, surpassing current UK intake of 1860.00 Kcal, 224.00 g carbs and 76.90 g protein [37], in line with NHS guidelines of at least 260.00 g of carbohydrates [38].

**Table 1:** Descriptive characteristics of the study population, mean (SD)

Phenotype	Mean	SD	
*N*	986		
M to F	277:726		
Age, years	45.65	11.96	
BMI, kg/m^2^	25.59	5.05	
*White blood cells*			
Basophils	0.05	0.05	
Eosinophils	0.14	0.11	
Lymphocytes	1.58	0.44	
Monocytes	0.41	0.13	
Neutrophils	3.10	1.19	
WBC	5.25	1.44	
*Dietary measures*			**% EI**
Energy, Kcal	2345.97	851.37	
Carbohydrate, g	262.36	100.37	44.70
Fat, g	109.19	48.09	41.90
Protein, g	80.75	28.54	13.80
Vegetable intake*, portions	5.55	2.91	
Fruit intake*, portions	2.76	1.90	
h-PDI score	59.48	7.31	
u-PDI score	59.23	6.68	
*Clinical measures*			
SBP, mmHg	109.85	12.87	
DBP, mmHg	74.10	8.70	
IL6	0.74	1.21	
Fasting glucose, mmol/L	7.55	1.13	
Glucose at 60 min, mmol/L	5.74	1.50	
Glucose at 120 min, mmol/L	5.45	1.27	
Insulin rise 30 min, mmol/L	60.19	51.26	
Insulin rise 120 min, mmol/L	31.24	27.58	
Fasting triglycerides, mmol/L	1.07	0.54	
Triglycerides at 360 min, mmol/L	1.90	1.20	

*Abbreviations*: *BMI* body mass index, *WBC* white blood cells, *EI* energy intake, *h-PDI* healthy plant-based diet index, *u-PDI* unhealthy plant-based diet index, *SBP* systolic blood pressure, *DBP* diastolic blood pressure, *IL6* interleukin-6, * energy adjusted

**Fig. 1:**
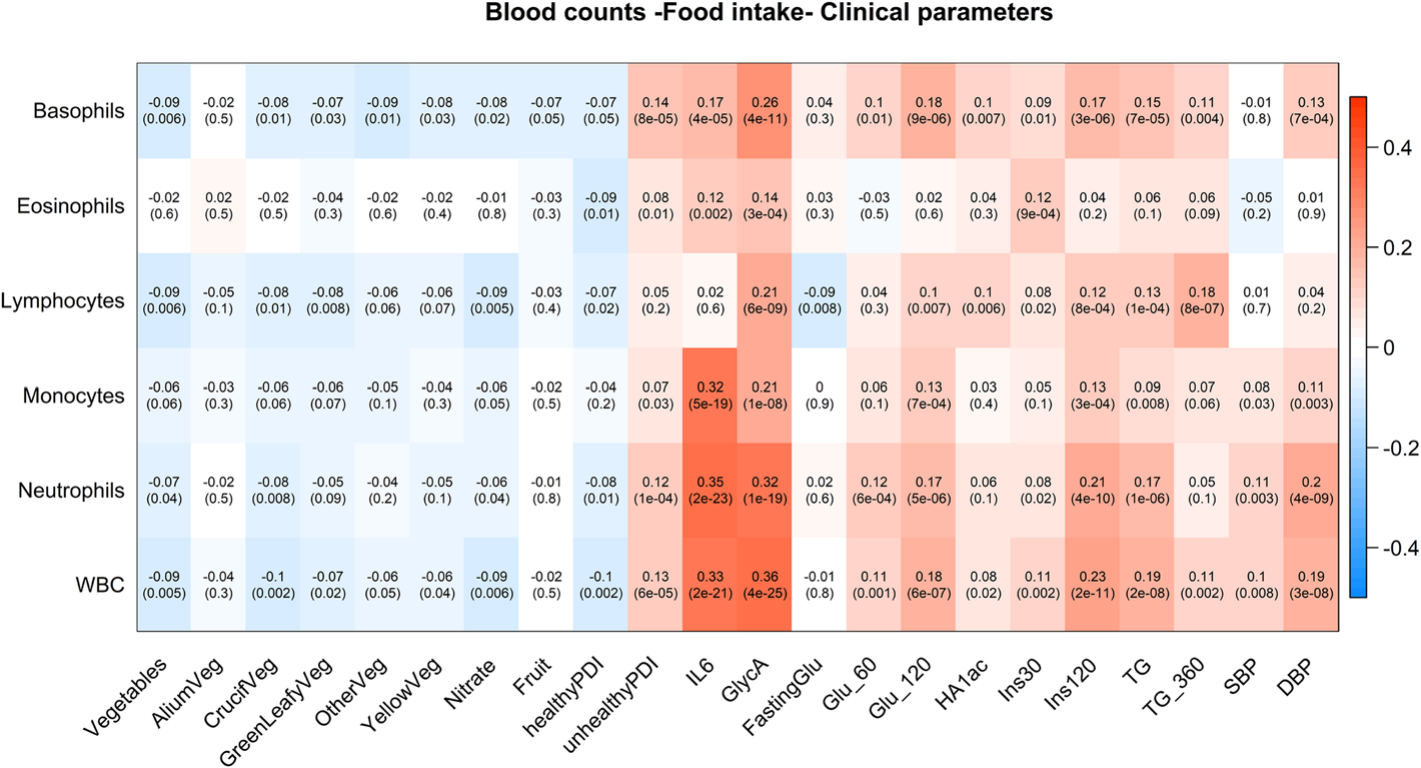
Each cell of the matrix contains the standardised regression coefficient between one white cell trait and a food item or clinical trait and the corresponding *p* value. The table is colour coded by correlation according to the table legend (red for positive and blue for negative correlations)

We found that WBC were positively correlated with higher postprandial glycaemic response, higher levels of the proinflammatory cytokine IL-6, higher GlycA (a marker of systemic inflammation and cardiovascular disease risk [39]), higher glycated haemoglobin and postprandial insulin, consistent with such counts being markers of chronic inflammation and cardiometabolic risk (Fig. 1). Lymphocytes, basophils and WBC counts were all significantly correlated with lower habitual intake of vegetables, with vegetable intake explaining between 3.59 and 6.58% of white blood cells (Fig. 1) after adjusting for age, gender, BMI and multiple testing using false discovery rate (*q* < 0.1). The results were consistent when further adjusting for family relatedness. No such association was observed with fruit intake (Fig. 1). We also found that (i) all WBC (but monocytes) are negatively correlated with having a healthy diet, measured via the Healthy PDI score [30], and (ii) all but lymphocytes are positively correlated with having an unhealthy diet, measured via the Unhealthy PDI score [30].

**Fig. 2:**
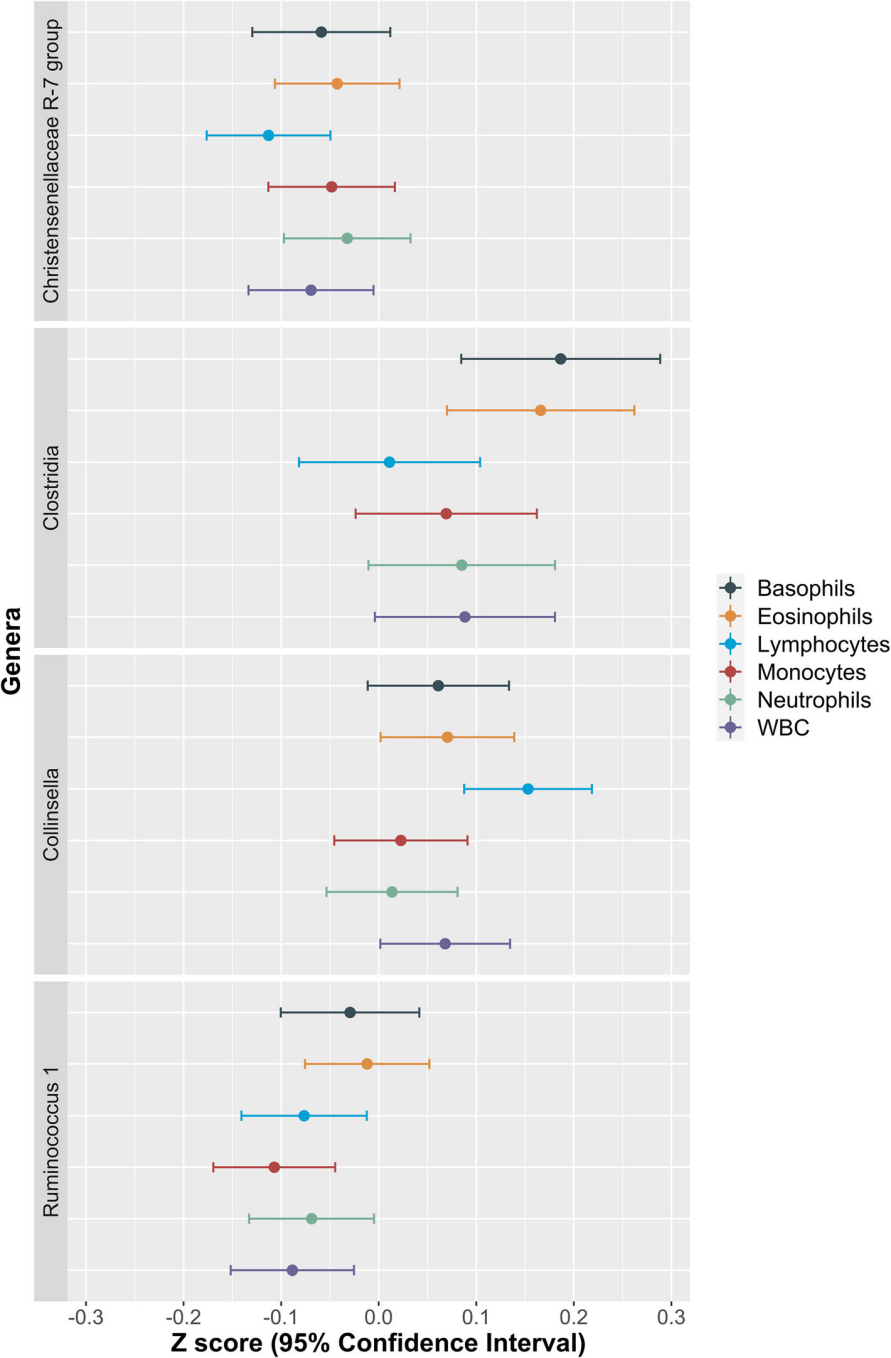
White blood cell–microbiome associations. Analyses adjusted by age, sex, BMI and multiple testing using FDR correction (*q* < 0.1)

We then examined the association between WBC and bacterial abundances (genera present in at least 70% of the sample). We identified 2 genera significantly associated with lymphocytes, one with basophils, one with eosinophils and one with monocytes after adjusting for age, gender, BMI and multiple testing using FDR correction (FDR < 0.1) (Fig. 2 and Supplementary Table 2). These include the following: (i) the positive correlation of lymphocytes and *Collinsella*, a microbe known to be linked to intake of processed foods and to increase risk of fatty liver [40, 41]; (ii) the positive correlation of both basophils and eosinophils with Clostridia; (iii) the negative correlation of lymphocytes with *Christensenellaceae_R-7_group*; and (iv) the negative correlation of monocytes with *Ruminococcus_1*.

In addition, we found that *Collinsella* abundances were positively correlated with both eosinophils and WBC and negatively correlated with vegetable intake as well as with nitrate intake.

**Fig. 3:**
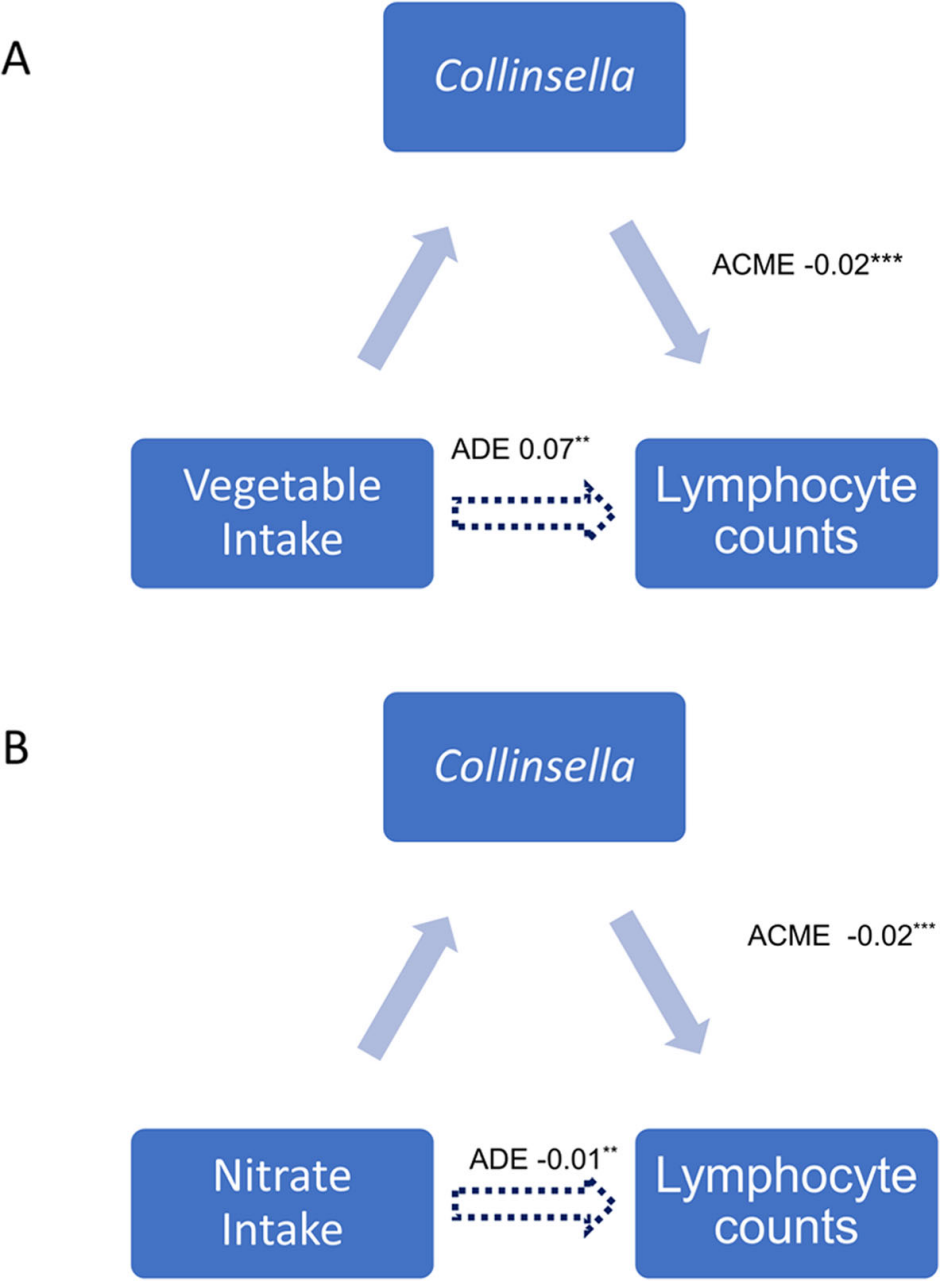
Mediation analysis of the association between (**a**) vegetable intake and (**b**) dietary nitrate and lymphocytes count. Average direct effect (ADE) and average causal mediation effects (ACME) are reported (*P < 0.05; **P < 0.01; ***P < 0.001)

We therefore conducted a formal mediation analysis to determine the indirect effect of the gut microbiome (*Collinsella*) on the effect between vegetable intake and lymphocytes. The analysis found that the *Collinsella* acted as potential partial mediator in the negative association between vegetable intake and lymphocytes (VAF = 20.00% [11.12%, 67.04%] *P* < 0.001) (Fig. 3) and in the negative association between nitrate intake and lymphocytes (VAF = 12.79[8.25; 28.15%] *P* < 0.0001).

## Discussion

Here, we investigated the relationship between white blood cells, habitual diet and gut microbiome composition. When we investigated links with habitual diet, we found that high vegetable intake, particularly green leafy and cruciferous vegetables, correlated with lower levels of white blood cell counts. No such relationship was seen with fruit intake, which may be a consequence of the relatively high sugar content of fruit compared to vegetables [42] and that a high sugar diet has been shown to be pro-inflammatory [42, 43]. On the other hand, it may be due to the presence of compounds common in vegetables but not in fruits. This would exclude plant-based food components such as dietary fibre or polyphenols, which are found both in fruit and vegetables [44] and tend to be higher in fruits. We hypothesised that it could be dietary nitrate content, which has been related to improved cardiometabolic outcomes [45], increased nitric oxide production and altered inflammation and immune function [18]. Approximately 60–80% inorganic nitrate exposure in the human diet is contributed by vegetable consumption [46]. Although inorganic nitrate is a relatively stable molecule, under specific conditions, it can be metabolised in the body to produce nitrous oxide (NO) via the nitrate–nitrite–NO pathway. NO is a major signalling molecule in the human body and has a key role in maintaining vascular tone, smooth muscle cell proliferation, platelet activity and inflammation [46, 47]. In animal models, dietary nitrate has been shown to attenuate endothelial dysfunction in animals fed a high-fat diet [48] or with diabetes [49], and has equivalent effects to those of metformin on glucose/insulin homeostasis and even larger effects regarding protection against cardiovascular dysfunction and liver steatosis [50]. We indeed find that nitrate content is also correlated with lower blood cell counts corresponding to lower cardiometabolic risk. Specifically, an increase in total WBC, lymphocytes, monocytes and neutrophil counts has been associated with higher CVD risk and total white blood cell count could potentially serve as a marker to predict CVD risk [51].

We then investigated the links between inflammatory cell profiles and gut microbiome composition and found that in particular the genus *Collinsella* was significantly associated with both lower vegetable intake and higher blood cell counts. In particular, the relative abundance of *Collinsella* explains 20% of the effect of vegetable intake on lymphocyte cell counts. The genus *Collinsella* has been previously reported to be significantly associated with higher risk of liver steatosis [40]. Moreover, its abundance in gnotobiotic mice increases with an increased intake of highly processed foods containing advanced glycation end products (AGEs) which are present in many heat-processed foods rich in starch, including potato chips and French fries [41]. Our data suggest that the abundance of *Collinsella* may be negatively affected by vegetable intake or nitrates. Nitric oxide production has been postulated as a possible molecular mechanism for the cardiometabolic risk reduction seen with the vegetable-rich Mediterranean diet [52]. Importantly, two *Collinsella* species were the key taxa whose abundances decrease as a result of a Mediterranean diet intervention in a 12-month intervention in 612 elderly participants across 5 European countries [53] further highlighting the link between the benefits of a high-nitrate vegetable-rich diet and low *Collinsella* abundance. Our results suggest that this may be enhanced or mediated by the lower abundance of the genus *Collinsella*, which in turn increases upon intake of processed foods. There have been previous studies that have shown the association of the microbiome with blood cell dynamics including associations with neutrophil to lymphocyte ratios that are correlated with the prognosis of multiple diseases including inflammatory and cardiovascular disease [13, 54, 55]. In the current study, we were able to infer for the association of the microbiota on systemic immune cell dynamics and how these could potentially be modulated via the diet.

We note some study limitations. First, this is an observational study with cross-sectional data and we have not carried out a vegetable intake intervention to assess its effect on pro-inflammatory cell count profiles. Future studies should investigate the causality of the observed decrease in white blood cell counts. Second, we have not been able to measure nitric oxide production in our samples as faecal samples were immediately processed for DNA extraction as per protocol and no supernatant for nitrates was therefore available. Third, we have used FFQs rather than other methods for assessing dietary intake. FFQs have become a well-accepted method for quantitative assessment of usual nutrient intake [54], but being recall data are subject to some bias. However, the value of FFQs for assessing dietary composition has been documented objectively by correlations with biochemical indicators and the prediction of outcomes in prospective studies [55, 56]. On the other hand, the data presented here links gut microbiome, cell counts and diet in a deeply phenotyped cohort and helps generate specific hypotheses to design dietary intervention studies aimed at reducing pro-inflammatory white cell profiles.

## Conclusion

Here, we link the gut microbiome, cell counts and diet in a deeply phenotyped cohort, setting a foundation to generate specific hypotheses to be tested in dietary intervention studies aimed at reducing pro-inflammatory white cell profiles. Our data highlight the link between vegetable dietary intake, reduced abundance of a bacterial genus, which increases with the intake of processed foods (Collinsella), and higher levels of immune-response cells involved in inflammatory processes. Very recently, the direct functional links between gut microbiome composition and immune response in humans [55] have been demonstrated in the 10-year follow-up of 2000 cancer patients. The next step, which our work contributes to address, is understanding the dietary patterns and specific nutrients involved in modifying the gut microbes that influence immune-response cells. Understanding such links can help develop dietary interventions to reduce inflammatory patterns involved in a vast array of pathophysiological processes from response to infections, to cancer and chronic cardiometabolic diseases.

## Supplementary Information

**Additional file 1: Table S1.** Vegetables aggregated to characteristic groups. **Table S2.** Standardised coefficients and 95%CI of FDR (*q* < 0.1) significant white blood cell and gut microbiome association adjusting for age, sex and BMI.

## Data Availability

The data used in this study are held by the department of Twin Research at KCL. The data can be released to bona fide researchers using our normal procedures overseen by the Wellcome Trust and its guidelines as part of our core funding (https://twinsuk.ac.uk/resources-for-researchers/access-our-data/). The 16S microbiome data will be uploaded onto the EBI website (https://www.ebi.ac.uk/) with unlimited access.

## Abbreviations

AGE: Advanced glycation end products
BMI: Body mass index
FFQ: Food frequency questionnaire
h-PDI: Healthy plant-based index
u-PDI: Unhealthy plant-based index
VAF: Variance accounted for
WBC: White blood cells
