# Supplementary material for: High intake of vegetables is linked to lower white blood cell profile and the effect is mediated by the gut microbiome
Source: BMC Med. 2021 Feb 11;19:37. doi: 10.1186/s12916-021-01913-w (PMC7875684; doi:10.1186/s12916-021-01913-w)
Supplement: Supplementary file 1 — Additional file 1: Table S1. Vegetables aggregated to characteristic groups. Table S2. Standardised coefficients and 95%CI of FDR (q < 0.1) significant white blood cell and gut microbiome association adjusting for age, sex and BMI. [file 12916_2021_1913_MOESM1_ESM.docx]

**Table S1. Vegetables aggregated to characteristic groups.**

| Vegetable grouping | Vegetables |
| --- | --- |
| Allium | Garlic, leeks, onions |
| Cruciferous | Broccoli, Brussel sprouts, cabbage, cauliflower, coleslaw |
| Green leafy | Green salad, spinach |
| Yellow | Carrots, tomatoes |
| Other | Avocado, beetroot, courgette, mushrooms, parsnip/turnip/swede, sweet peppers, sweetcorn, watercress. |
| Total | All groups, vegetable soup |

**Table S2. Standardised coefficients and 95%CI of FDR (q<0.1) significant white blood cell and gut microbiome association adjusting for age, sex and BMI.**

| Genus | WBC | Beta | 95%CI | P | Q |
| --- | --- | --- | --- | --- | --- |
| Collinsella | lymphocytes | 0.15 | [0.09;0.22] | 5.29E-06 | 2.70E-03 |
| Christensenellaceae_R-7_group | lymphocytes | -0.11 | [-0.18;-0.05] | 5.10E-04 | 0.08 |
| Ruminococcus_1 | monocytes | -0.11 | [-0.17;-0.04] | 8.11E-04 | 0.08 |
| Clostridia | basophils | 0.19 | [0.08;0.29] | 3.83E-04 | 0.08 |
| Clostridia | eosinophils | 0.17 | [0.07;0.26] | 7.75E-04 | 0.08 |
